# Group B Streptococcal Disease Worldwide for Pregnant Women, Stillbirths, and Children: Why, What, and How to Undertake Estimates?

**DOI:** 10.1093/cid/cix653

**Published:** 2017-11-06

**Authors:** Joy E Lawn, Fiorella Bianchi-Jassir, Neal J Russell, Maya Kohli-Lynch, Cally J Tann, Jennifer Hall, Lola Madrid, Carol J Baker, Linda Bartlett, Clare Cutland, Michael G Gravett, Paul T Heath, Margaret Ip, Kirsty Le Doare, Shabir A Madhi, Craig E Rubens, Samir K Saha, Stephanie Schrag, Ajoke Sobanjo-ter Meulen, Johan Vekemans, Anna C Seale

**Affiliations:** 1 Maternal, Adolescent, Reproductive and Child Health Centre, London School of Hygiene & Tropical Medicine, United Kingdom; 2 King’s College London, United Kingdom; 3 Centre for Child and Adolescent Health, School of Social and Community Medicine, University of Bristol, United Kingdom; 4 Neonatal Medicine, University College London Hospitals NHS Foundation Trust, United Kingdom; 5 Department of Reproductive Health Research, University College London Institute for Women’s Health, United Kingdom; 6 ISGlobal, Barcelona Centre for International Health Research, Hospital Clinic–University of Barcelona, Spain; 7 Departments of Pediatrics and Molecular Virology and Microbiology, Baylor College of Medicine, Houston, Texas; 8 Department of International Health, Johns Hopkins Bloomberg School of Public Health, Baltimore, Maryland; 9 Medical Research Council: Respiratory and Meningeal Pathogens Research Unit, and Department of Science and Technology/National Research Foundation: Vaccine Preventable Diseases, University of the Witwatersrand, Faculty of Health Sciences, Johannesburg, South Africa; 10 Global Alliance to Prevent Prematurity and Stillbirth; 11 Department of Obstetrics and Gynecology, University of Washington, Seattle; 12 Vaccine Institute, Institute for Infection and Immunity, St George’s Hospital, University of London and St George’s University Hospitals NHS Foundation Trust, United Kingdom; 13 Department of Microbiology, Faculty of Medicine, Chinese University of Hong Kong; 14 Centre for International Child Health, Imperial College London, United Kingdom; 15 National Institute for Communicable Diseases, National Health Laboratory Service, Johannesburg, South Africa; 16 Department of Global Health, University of Washington, Seattle; 17 Bangladesh Institute of Child Health, Dhaka; 18 National Center for Immunization and Respiratory Diseases, Centers for Disease Control and Prevention, Atlanta, Georgia; 19 Bill & Melinda Gates Foundation, Seattle, Washington; 20 World Health Organization, Geneva, Switzerland; 21 College of Health and Medical Sciences, Haramaya University, Dire Dawa, Ethiopia

**Keywords:** group B *Streptococcus*, global burden, stillbirth, neonatal, maternal

## Abstract

Improving maternal, newborn, and child health is central to Sustainable Development Goal targets for 2030, requiring acceleration especially to prevent 5.6 million deaths around the time of birth. Infections contribute to this burden, but etiological data are limited. Group B *Streptococcus* (GBS) is an important perinatal pathogen, although previously focus has been primarily on liveborn children, especially early-onset disease. In this first of an 11-article supplement, we discuss the following: (1) Why estimate the worldwide burden of GBS disease? (2) What outcomes of GBS in pregnancy should be included? (3) What data and epidemiological parameters are required? (4) What methods and models can be used to transparently estimate this burden of GBS? (5) What are the challenges with available data? and (6) How can estimates address data gaps to better inform GBS interventions including maternal immunization? We review all available GBS data worldwide, including maternal GBS colonization, risk of neonatal disease (with/without intrapartum antibiotic prophylaxis), maternal GBS disease, neonatal/infant GBS disease, and subsequent impairment, plus GBS-associated stillbirth, preterm birth, and neonatal encephalopathy. We summarize our methods for searches, meta-analyses, and modeling including a compartmental model. Our approach is consistent with the World Health Organization (WHO) Guidelines for Accurate and Transparent Health Estimates Reporting (GATHER), published in *The Lancet* and the Public Library of Science (PLoS). We aim to address priority epidemiological gaps highlighted by WHO to inform potential maternal vaccination.

## Teaser

Group B *Streptococcus* is associated with multiple adverse pregnancy outcomes, yet previous epidemiology and interventions have mainly focused on neonatal invasive disease in high-income settings. We describe the rationale and approach for the first worldwide estimates of all relevant outcomes.

Despite remarkable progress for child survival during the Millennium Development Goal (MDG) era to 2015 [1], halving deaths for children aged <5 years, still an estimated 5.9 million children die per year. Almost half (45%) of these deaths are in the first month of life (neonatal period), where investment and progress has been much slower [2, 3]. In addition to the 2.7 million neonatal deaths, an estimated 2.6 million third-trimester stillbirths occur each year, but are often left out of impact and cost-effectiveness analyses [4]. More innovation and investment are required to reduce these 5.3 million deaths, plus 0.3 million maternal deaths, which also occur around the time of birth.

The Sustainable Development Goals (SDGs) aim to end preventable maternal and child deaths by 2030 [5], and include the first ever target for newborns, aiming that every country should have ≤12 neonatal deaths per 1000 livebirths by 2030 (Table 1) [2, 6]. Because these targets are national, the countries with the highest mortality risk now, which are mostly in Africa, will have to make major shifts in their rates of mortality reduction. For example, 49 countries need to at least double their current average annual reduction for neonatal mortality rates to meet 2030 targets [7]. Investments should be prioritized based on the best epidemiological data, including more detailed etiology of infectious causes, and prioritizing strategies that are more likely to reach the poorest families where most of these deaths occur.

**Table 1.** Progress for Ending Preventable Deaths for Women, Neonates, Children, and Stillbirths

	Maternal Deaths	Stillbirths	Neonatal Deaths	Child Deaths (0–59 mo, Including Neonatal)
Global numbers of deaths during the Millennium Development Goal era (1990–2015) [1]				
1990	0.53 million	Not available	5.1 million	12.7 million
2000	0.44 million	3.2 million	3.9 million	9.8 million
2015	0.33 million	2.6 million	2.7 million	5.9 million
Targets for the Sustainable Development Goal era from 2016 to 2030 [5]				
Target	Every country should reduce its maternal mortality ratio by at least two-thirds from the 2010 baseline, and no country should have a rate >140 deaths per 100000 live births (twice the global target). The global average^a^ target of maternal mortality ratio should be <70 maternal deaths per 100000 live births	Every country should have a stillbirth rate of ≤12 per 1000 total births. This would result in an average global neonatal mortality rate of 9 per 1000 total births.	Every country should have a national neonatal mortality rate of ≤12 per 1000 live births. This would result in an average global neonatal mortality rate of 9 per 1000 live births.	Every country should have a national under-5 mortality rate of ≤25 per 1000 live births. This would result in an average global under-5 mortality rate of 17.2 per 1000 live births.
Action plan or strategy linked to Sustainable Development Goals	Ending preventable maternal mortality Global Strategy for Women’s, Children’s and Adolescents’ Health [61]	Every Newborn Action Plan Global Strategy for Women’s, Children’s and Adolescents’ Health [62]		A Promise Renewed Global Strategy for Women’s, Children’s and Adolescents’ Health [63]
Number of deaths in 2030 if target is met^a^	Not estimated	1.1 million	0.8 million	2.4 million
Number of countries to at least double rate of progress	Not estimated	56	49	19

^a^Assuming same average annual rate of mortality reduction (2000–2015), while taking account of predicted national demographic change.

Sources: World Health Organization (WHO), United Nations Children’s Fund (UNICEF), United Nations Population Fund, World Bank Group, United Nations Population Division. Trends in maternal mortality: 1990 to 2015.

Lawn JE, et al [2].

Lawn JE, et al [7].

WHO, UNICEF [62].

United Nations Interagency Group for Child Mortality Estimation. Levels and Trends in Child Mortality 2015.

**Figure 1.**
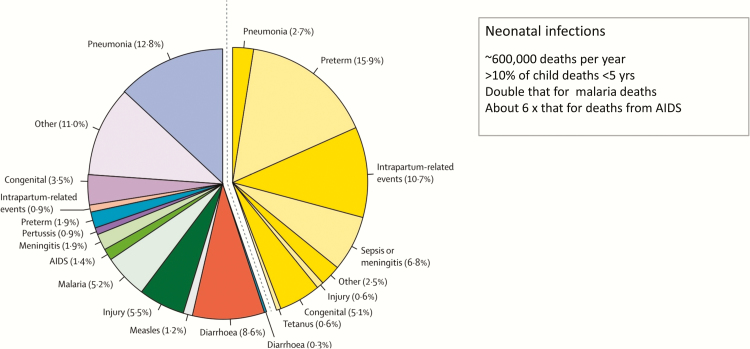
Causes of deaths for neonates and children aged <5 years in 2015. Source: Liu et al [64].

Worldwide from 2000 to 2015, 9 of the 10 most rapidly reducing causes of child death were infections [8]. The fastest progress has been made for AIDS deaths in children, reducing at 6.7% per year and now down to 103000 deaths. Crucial to this rapid progress were disease burden estimates for all countries, and targeted interventions with drugs or vaccines, with coverage data to monitor progress. Data are critical for public health decision making, to prioritize investment in the largest-burden conditions affecting the poorest populations. Yet while the poorest and most vulnerable populations have the highest risk of most diseases, they also have the least data—the “inverse data law.” This particularly applies to the estimated 600000 child deaths due to neonatal infections, which is more than that for malaria and AIDS combined (Figure 1). Yet data are lacking regarding the etiology of these deaths. The current global intervention strategy is to use sensitive but nonspecific algorithms to identify possible serious bacterial infection and then to treat all these neonates and infants with antibiotics [9, 10]. With improved diagnostics, targeted treatment could be delivered to support care, and reduce use of broad-spectrum antibiotics that select for antimicrobial resistance. With improved etiological data, targeted interventions, such as prevention by immunization, may also be possible.

Group B *Streptococcus* (GBS), or *Streptococcus agalactiae*, is a β-hemolytic gram-positive coccus. It can be part of the normal human and animal microflora, and was first identified as a pathogen in animals, causing bovine mastitis, in 1887 [11]. GBS was subsequently identified as a human pathogen causing puerperal sepsis in London, United Kingdom, in 1938 [12]. Later, GBS emerged as an important cause of neonatal septicemia and meningitis in the United States, with cases increasing from the 1960s [13, 14], followed by increases in other high-income contexts, such as the United Kingdom, by the 1980s [15]. The reasons for the emergence of GBS are unclear; theories have included the mechanization of dairy farming increasing the spread of GBS [16], a species jump from bovines [17], and/or the spread of a virulent GBS clone [18, 19], possibly related to the development of tetracycline resistance, with its widespread use [20].

In this article, the first of 11 covering the most comprehensive assessment to date of data regarding disease burden of GBS, we address 6 questions that guide the methodological approach taken throughout the supplement (Table 2).

**Table 2.** Group B *Streptococcus* Estimates and Questions to Be Addressed to Inform the Methodological Approach Applied

1. Why estimate the worldwide burden of group B *Streptococcus* (GBS) disease?
2. What outcomes of GBS should be considered in estimates?
3. What data and epidemiological parameters are therefore required?
4. What methods and models can be used to transparently estimate this burden of GBS?
5. What are the challenges with the available data?
6. How can estimates address data gaps to better inform GBS interventions including maternal immunization?

## QUESTION 1. WHY ESTIMATE THE WORLDWIDE BURDEN OF GROUP B *STREPTOCOCCUS* DISEASE?

In high-income contexts, where there is good capture of cases and routine laboratory surveillance, *S. agalactiae* or GBS is now well-recognized as one of the leading cause of infant deaths, particularly in the early neonatal period (first week). Strategies of intrapartum antibiotic prophylaxis have been applied to address this burden, notably early-onset disease. GBS is also a candidate for maternal vaccine development.

However, there remains uncertainty regarding the geographic distribution of GBS and the reasons why large etiology studies in low- and middle-income contexts in the 1990s [21, 22] and more recently [23] have not identified GBS, whereas facility studies from some of the same countries, notably in South Africa, Kenya, and The Gambia, reported much higher incidence [24–26]. There are particular uncertainties in South Asia, where reported differences may be real, or at least partly explained by differences in case ascertainment. Gram-negative infections dominate in both facility-based [27] and community-based [28, 29] studies. Especially in some South Asian settings where most births are at home, and given the high case fatality with GBS, deaths may occur before reaching a facility or before community workers come to the home [25]. In addition, the use of peripartum antibiotics over the counter (which is also very high in South Asia) could reduce detection and/or GBS disease. Hence, regional differences may be due to challenges in case ascertainment, or they may be true epidemiological and microbiological variation linked to the emergence of GBS disease or regional differences in virulence—for example, higher prevalence of the most virulent clone, usually associated with serotype III [30].

Estimating the burden of disease informs global public health policy, exemplified by the annual global burden of disease estimates for 310 diseases and injuries [31]. Systematic and transparent estimates of the worldwide burden of GBS disease are required to guide investment in interventions, and specifically to be able to assess the potential value of candidate GBS maternal vaccines.

**Figure 2.**
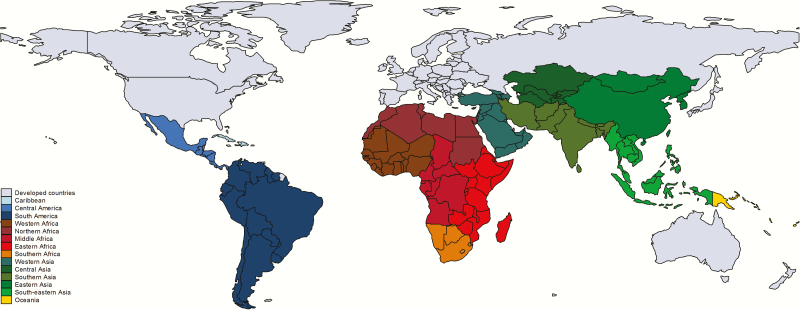
Map of United Nations subregions that will be used for reporting input data and results. Borders of countries/territories in map do not imply any political statement.

Therefore, we have made extensive attempts to access all data available from as many countries as possible—published and also unpublished—in collaboration with investigators worldwide. We report input data and results for the United Nations subregions shown in Figure 2.

## QUESTION 2. WHAT OUTCOMES OF GROUP B *STREPTOCOCCUS* MATERNAL COLONIZATION SHOULD BE CONSIDERED IN ESTIMATES?

**Figure 3.**
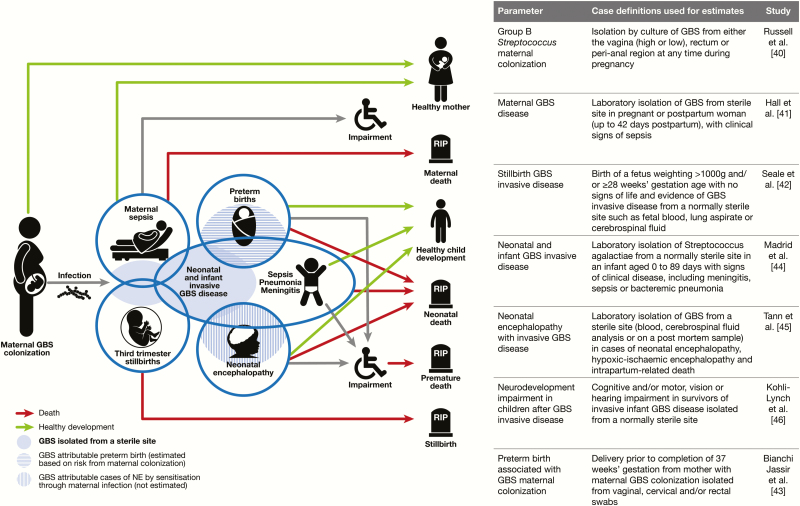
Disease schema for outcomes of perinatal group B *Streptococcus.* Abbreviations: GBS, group B *Streptococcus*; NE, neonatal encephalopathy.

For the half-century history of GBS, most focus has been on infant invasive disease, particularly early-onset disease in the early neonatal period (first week); including how to identify, treat, and then how to prevent, primarily with intrapartum antibiotic prophylaxis. While early-onset disease is an important consequence of maternal GBS colonization, a focus only on neonatal and infant disease has missed other important outcomes and contributors to the burden of GBS disease (Figure 3).

Learning from other global health conditions, the woman should be included in her own right as well as to improve outcomes for her child [32]. Although puerperal sepsis was the first clinical syndrome in which GBS was identified as a human pathogen, there have been limited reviews focused on maternal GBS disease.

GBS-associated stillbirths are also rarely considered. Stillbirths are often not included in global monitoring data systems [7] due to the stigma, which is seen even in high-income contexts, and fatalism regarding prevention [33]. It was not until 2017 that the World Health Organization (WHO) officially asked countries for stillbirth data, alongside other mortality data reporting. Not counting stillbirths is misleading in terms of the total burden of GBS disease, and from a family and society perspective. The death of an infant in the last weeks of pregnancy, or after birth, is a catastrophic event [34]. GBS is a cause of stillbirth and, although data are limited, recent studies from Kenya and South Africa are available [25, 35].

The classic invasive GBS disease syndromes of sepsis and meningitis may overlap with other leading causes of neonatal death, such as neonatal encephalopathy. Globally hypoxic insult is the most common cause of neonatal encephalopathy [36], but infection exposure likely increases the risk of hypoxic damage. To date, very few studies have examined the proportion of cases of neonatal encephalopathy that are culture positive, including for GBS.

In addition, while preterm birth is a known risk factor for invasive GBS disease, several studies have suggested that maternal GBS colonization may increase the risk of preterm birth. However, published data are somewhat contradictory and may inappropriately combine different study designs [37]. The WHO and others have highlighted this as a priority for more analysis [38], especially now that preterm birth is the leading cause of under-5 deaths (Figure 1).

Nonfatal outcomes, particularly impairment and associated disability, have consequences for families and societies. The Global Burden of Disease study underlines that as mortality reduces, the risk of disability among survivors may actually form a greater burden than the deaths, and impairment is an important consequence of neonatal infection [39]. As GBS is a leading contributor to neonatal infection, its contribution to this should also be assessed.

Therefore, in this exercise we aim to consider all the relevant outcomes from GBS colonization in pregnant women [40], maternal GBS disease [41], stillbirths [42], and preterm births [43] associated with GBS, neonatal and infant GBS disease [44], GBS-associated neonatal encephalopathy [45], and impairment after neonatal/infant GBS disease [46]. These outcomes are shown in a disease schema (Figure 3) indicating the main pathway of mother-to-child transmission, and some of the potential overlaps, for example, between preterm birth and GBS disease in neonates.

The case definitions for the main outcomes of interest are in Figure 3; for each we sought a definition including GBS isolation from a sterile site, knowing that this is conservative and may undercount cases, as discussed below.

## QUESTION 3. WHAT INPUT DATA AND EPIDEMIOLOGICAL PARAMETERS ARE THEREFORE REQUIRED?

The most important principle is to maximize the available data, applying explicit inclusion and exclusion criteria. The lack of systematic surveillance data, especially for the highest-burden countries, means that modeling is inevitable for worldwide estimates. Given the complexity of methods and variable reporting approaches, there has been an erosion of public trust in estimates [47]. Hence, to promote transparency, WHO with the Institute of Health Metrics and Evaluation, Seattle, and some independent experts including some authors on this series, have published the Guidelines for Accurate and Transparent Health Estimates Reporting (GATHER), as a standard for reviewing data inputs, biases, and reporting methods [48]. The articles in this supplement follow the GATHER checklist through the process from data assessment to final publication, including open access data and code (Table 3).

**Table 3.** Guidelines for Accurate and Transparent Health Estimates Reporting (GATHER)

**Item #**	**Checklist Item**	**Reported in Paper** No.
Objectives and funding		
1	Define the indicator(s), populations (including age, sex, and geographic entities), and time period(s) for which estimates were made.	All papers
2	List the funding sources for the work.	All papers
Data inputs		
For all data inputs from multiple sources that are synthesized as part of the study:		
3	Describe how the data were identified and how the data were accessed.	All papers
4	Specify the inclusion and exclusion criteria. Identify all ad hoc exclusions.	All papers
5	Provide information on all included data sources and their main characteristics. For each data source used, report reference information or contact name/institution, population represented, data collection method, year(s) of data collection, sex and age range, diagnostic criteria or measurement method, and sample size, as relevant.	All papers
6	Identify and describe any categories of input data that have potentially important biases (eg, based on characteristics listed in item 5).	All papers
7	Describe and give sources for any other data inputs.	All papers
8	Provide all data inputs in a file format from which data can be efficiently extracted (eg, a spreadsheet rather than a PDF), including all relevant metadata listed in item 5. For any data inputs that cannot be shared because of ethical or legal reasons, such as third-party ownership, provide a contact name or the name of the institution that retains the right to the data.	All papers
Data analysis		
9	Provide a conceptual overview of the data analysis method. A diagram may be helpful.	All papers
10	Provide a detailed description of all steps of the analysis, including mathematical formulae. This description should cover, as relevant, data cleaning, data preprocessing, data adjustments and weighting of data sources, and mathematical or statistical model(s).	All papers
11	Describe how candidate models were evaluated and how the final model(s) were selected.	[1, 2, 11]
12	Provide the results of an evaluation of model performance, if done, as well as the results of any relevant sensitivity analysis.	[2, 11]
13	Describe methods for calculating uncertainty of the estimates. State which sources of uncertainty were, and were not, accounted for in the uncertainty analysis.	[1, 11]
14	State how analytic or statistical source code used to generate estimates can be accessed.	[2, 11]
Results and discussion		
15	Provide published estimates in a file format from which data can be efficiently extracted.	[11]
16	Report a quantitative measure of the uncertainty of the estimates (eg, uncertainty intervals).	[11]
17	Interpret results in light of existing evidence. If updating a previous set of estimates, describe the reasons for changes in estimates.	[1, 11]
18	Discuss limitations of the estimates. Include a discussion of any modeling assumptions or data limitations that affect interpretation of the estimates.	[1, 11]

Source: [48].

To maximize data inputs, we review all published literature on GBS worldwide, applying prespecified criteria and case definitions (Figure 3). Databases searched include Medline, Embase, the WHO library database (WHOLIS), Scopus, and Literature in the Health Sciences in Latin America and the Caribbean (LILACS). For each paper, the particular search for GBS disease outcome is given according to international guidelines [49]. In all papers we used Medical Subject Heading (MeSH) terms related to GBS: Streptococcus OR Streptococcal OR Streptococci AND (Group AND B) or agalactiae; *Streptococcus agalactiae*. When needed, secondary analyses were requested from authors. In addition, searches of trial and study registries were undertaken and investigators were approached. Data were abstracted by at least 2 people and assessed for biases as described in each of the relevant papers. Biases that apply to GBS data generally are discussed below and, where specific to a given parameter, are covered in the relevant paper. Meta-analyses were undertaken using random-effects modeling to estimate pooled measures of effect using the DerSimonian and Laird method [50].

## QUESTION 4. WHAT MODELS CAN BE USED TO TRANSPARENTLY ESTIMATE THIS BURDEN OF GROUP B *STREPTOCOCCUS*?

Modeling complexity increased during the MDG era, exemplified by the Global Burden of Disease project, where the number of outcomes, the modeling complexity, and the time load increased markedly [37]. Here we will not attempt to summarize the plethora of statistical modeling methodologies, but briefly summarize some methodological options for estimating the worldwide burden of GBS disease.

For GBS, as with most infections, there is not just one parameter but multiple outcomes even in one individual, and the aim is to predict this mix of outcomes at the population level. The most well-known approaches for infectious disease modeling focus on epidemic conditions [51], where transmission rates are high, and are based on a dynamic infection compartmental model. The simplest of these is a 3-compartment SIR model as follows: S = number susceptible, I = number infectious, and R = number recovered (immune). For epidemiological exposures around the time of birth, which are either noninfections (eg, hypoxia) or where the infection is mainly passed from mother to child (including GBS), then the main factors affecting cases are the risk at birth and demographic factors affecting births. In this case, a stable compartment model is appropriate and has been used for other estimates of perinatal outcomes [52] and operates in 4 steps as follows:

### Step 1. Exposure

For a given condition, what is the exposure prevalence at the population level (eg, an infection among pregnant women, or a blood group type such as rhesus negative)?

### Step 2. Cases

For exposed pregnant women, risk data are required to predict adverse birth outcomes such as stillbirths or preterm births. If these risks vary in different geographies or with other comorbidities (eg, human immunodeficiency virus [HIV]), then population-specific data would also be required as to how much these conditions prevail in the population and how they affect the risk.

### Step 3. Deaths

The number of deaths can be estimated from the number of cases, given adequate case fatality risk data, and the number of maternal deaths from the maternal cases, or neonatal deaths from the neonatal cases.

### Step 4. Impaired Survivors

A final step can then predict the risk of neurodevelopmental impairment among survivors.

In the case of GBS, a stable compartmental approach is the best method to achieve the estimates of deaths and disability. This can be developed either sequentially or by applying Bayesian modeling, such as used in the Global Burden of Disease study [53]. A multiple regression model could be an option to estimate the prevalence of maternal GBS colonization by country, predicting the national prevalence based on national covariates, as an alternative to using reported data by country, or subregion [40]. We explore and report this option, which depends on successful model fitting [54].

A compartmental modeling approach to estimate the worldwide burden of GBS would require the following parameters for the 4 steps:

### Step 1. Exposed: Maternal Colonization With Group B *Streptococcus*

For the first step of the compartmental model, we begin with estimates of live births in 195 countries, and apply maternal GBS colonization prevalence for each country or, if not available, then meta-analysis for the relevant region

### Step 2. Cases of Group B *Streptococcus*

For the exposed population of pregnant women in each country, risk data would be required to predict the number of cases of GBS associated with GBS maternal colonization for each of the following outcomes: neonatal/infant invasive GBS disease, neonatal encephalopathy with GBS invasive disease, maternal sepsis, stillbirth, and preterm birth. To adjust these risks, we would also require population-specific data on variables affecting risk such as policy/coverage for intrapartum antibiotic prophylaxis, and how much the risk is reduced (or increased).

However, for some of these desired risk parameters, the compartmental model approach is not feasible. For example, research reporting eliminate stillbirths is more recent [7] and GBS associated stillbirths is usually reported as a proportion of stillbirths with GBS in a sterile site, rather than risk given maternal GBS colonization [42]. Similarly, incidence and risk data are rarely available for maternal disease, or neonatal encephalopathy [45] or preterm birth rate [43]. Hence, as detailed in papers 3, 4, and 5, the parameters sought were the incidence of GBS in a sterile site (Figure 3). To estimate the cases, this incidence is applied at a country level to the relevant denominator, which is national births in 2015 (for maternal GBS disease and GBS-associated neonatal encephalopathy) or to the specific denominator (ie, stillbirths or preterm births by country in 2015).

### Step 3. Deaths

Based on adequate data for case fatality rates, the number of neonatal/infant deaths can be estimated from the neonatal/infant cases, and the number of maternal deaths from the maternal cases. Challenges with accurate, population-based case fatality risk data are an important limitation in most compartmental models, whether stable or dynamic.

### Step 4. Impaired Survivors

Finally, the risk of neurodevelopmental impairment is applied to the number of GBS survivors per country, to estimate the number of children with disability. This step requires data on risk of impairment after GBS disease in neonates/infants, which is best derived from cohort studies.

**Figure 4.**
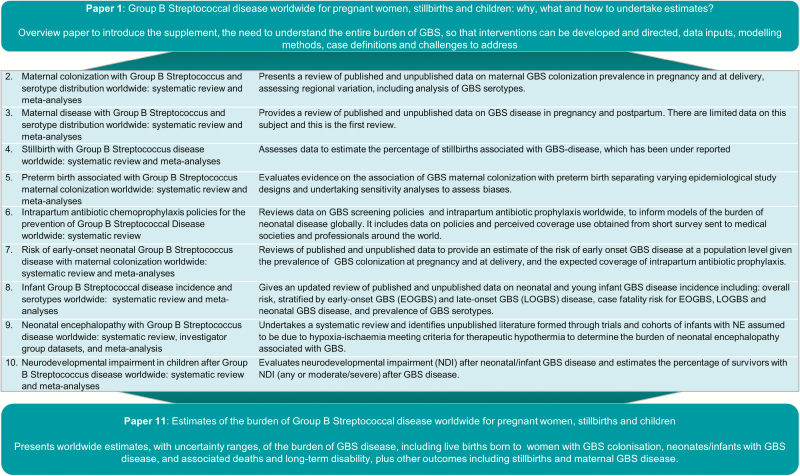
Overview of the articles in this supplement to estimate the worldwide burden of group B *Streptococcus*. Abbreviations: EOGBS, early-onset group B *Streptococcus*; GBS, group B *Streptococcus*; LOGBS, late-onset group B *Streptococcus*; NE, neonatal encephalopathy; NDI, neurodevelopmental impairment.

Based on the outcomes to estimate and the parameters required (Figure 3), the following 9 articles in this series will describe the case definitions and data available (Figure 4). The final article will provide details of the estimation methods. Uncertainty estimates are made, which is highlighted as an imperative in the GATHER statement [48].

As stated by Lord George Box, “All models are wrong, but some are useful” [55]. Based on this principle, we will undertake sensitivity analyses in each article regarding the key parameters being used for estimation, and we will also triangulate results where possible, for example, comparing the number of cases worldwide and by region and country for neonatal invasive disease as found in published literature compared to predicted, using the compartmental model outputs.

## QUESTION 5. WHAT ARE THE CHALLENGES WITH THE AVAILABLE DATA?

Modeling cannot overcome lack of data or very biased data. With respect to these data gaps and data biases, transparency is critical, and an important principle in GATHER is to recognize and describe biases [48].

**Figure 5.**
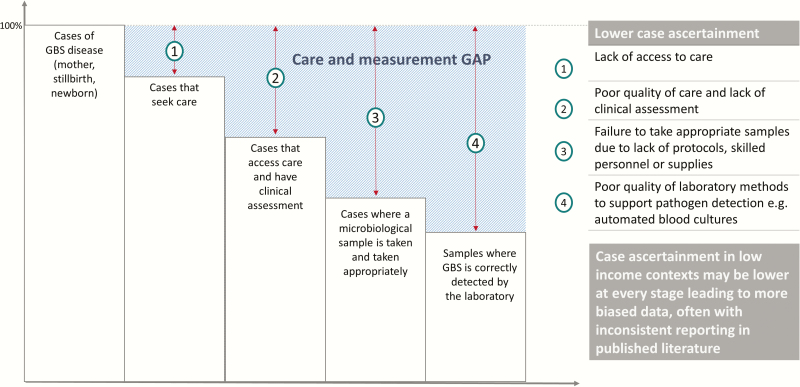
Data cascade for GBS disease showing the gap for care and measurement and the biases at added at each step. Adapted from Fitchett et al [59] and applied to the framework of the human immunodeficiency virus identification and treatment cascade. Abbreviation: GBS, group B *Streptococcus*.

In terms of GBS disease (whether in women or children), case ascertainment reduces at each stage of the care cascade [56], introducing measurement gaps and therefore biases that affect accuracy (Figure 5).

### Cases That Seek Care

In settings where most births are at home, the majority of early-onset cases may be missed. For example, in some parts of South Asia, in Ethiopia, and in northern Nigeria, 90% of births may be at home. Globally this may be the single greatest source of bias in the data, often differentially missing cases in the poorest settings. In our estimates, we will take into account for each country the proportion of births that are at home, since cases among home births are least likely to access care, and most likely to die uncounted.

### Cases That Are Assessed

In settings where quality of care in hospitals is lacking for neonates and sick young infants, cases may not be assessed, or infants may die before being effectively examined or managed. In South Africa, although >95% of births are in hospitals, the reported incidence for GBS early-onset disease based on passive surveillance from across all provinces varied from 0.00 to 1.23/1000 live births, and 0.03 to 1.04/1000 live births for late-onset disease [57].

### Cases That Have an Appropriate Microbiological Specimen Taken

Even where treatment is delivered, only a small proportion of hospital admissions may have investigations. An example is The Gambia, where 99% of neonates admitted with suspected infection did not have a blood culture and even fewer had a lumbar puncture [58]. Considering this bias, we will use risk data from settings with complete case ascertainment and appropriate investigation. The proportion of the world’s 2.6 million stillbirths that have a microbiological specimen taken is tiny [7]. In our estimates, we use data from studies where most stillbirths identified were investigated for GBS, so within these datasets the internal bias is lower.

### Specimens That Are Appropriately Processed in the Laboratory

In many low-resource contexts, laboratories are only open a few hours a day or have limited skilled staff or microbiological culture facilities, notably for blood culture. Detection by culture is also affected by previous antibiotic treatment, particularly where there is widespread use of “over the counter” antibiotics.

At each point along this cascade, the reduction in case ascertainment decreases the observed incidence of GBS disease, and introduces more bias, and those biases are greatest in low-resource settings. Therefore, in this exercise we aim to do all that is possible to minimize these biases, or, where this is not possible, describe and analyze the direction of bias, as follows:

• Increase the input data from as many countries as possible, aiming to use national-level data if adequate, otherwise pooling by relevant subregion (Figure 2).
• Collate details for each study/dataset regarding context of care seeking, case definitions, and laboratory methods, to allow assessment of case ascertainment and bias.
• Adjust where biases are predictable (eg, low sensitivity of laboratory detection due to method used) and report both adjusted and unadjusted data.
• Apply sensitivity analyses to examine the effect of different biases in the data, including varying case definitions.
• Compare estimates from the model with those reported from countries with complete or very high case ascertainment.

This examination of the available data also provides insights on how to improve research and routine data collection regarding GBS. Standardized reporting is critical, as described for neonatal infections in the Strengthening Reporting of Observational Studies in Epidemiology–Neonatal Infections (STROBE-NI) checklist [59] and case definitions, with the Brighton Collaborative regarding maternal immunization being especially relevant [60].

## QUESTION 6. HOW CAN ESTIMATES ADDRESS DATA GAPS TO BETTER INFORM GROUP B *STREPTOCOCCUS* INTERVENTIONS INCLUDING MATERNAL IMMUNIZATION?

The potential for maternal vaccines to use in high-, middle- and low-income contexts has been highlighted by WHO. The value proposition of new vaccines should be based on data. As part of a WHO-sponsored technical roadmap regarding GBS vaccine development to facilitate decision making by funders, vaccine researchers, and industry, improved disease burden and potential public health impact estimates have been highlighted as an important priority [38]. Based on WHO’s scoping, we have prioritized the following data gaps to address in this series of articles:

### Geographic Data From as Many Countries as Possible

This scoping stated that “the most important gap identified was regarding availability and quality of data on disease burden, and notably the limited information so far from some of the world’s poorest regions” [38]. Therefore, in this exercise we have made extensive attempts to identify data from as many countries as possible, also involving investigator groups and calls for unpublished data through regional and global networks. The details are provided in each article as relevant.

### Total Burden With All Relevant Outcomes of Group B *Streptococcus* Disease for Pregnant Women, Stillbirths, and Children

The potential role of vaccines to impact stillbirth and prematurity, and women, as well as to reduce long-term complications of invasive infections are major drivers of the estimated health and economical vaccine impact.

### Serotype Data to Inform Possible Regional Risk Variation and Vaccine Design

There were no published systematic assessments of GBS serotypes worldwide. Differences in geographical distribution of specific bacterial serotypes and strains need to be determined to guide optimal selection of vaccine targets, and this may also help to explain reported regional variation in GBS invasive disease. A future vaccine will need to overcome bacterial diversity of capsular polysaccharide serotype or target protein polymorphism. The characterization of virulence factors and frequency of capsular switching are important considerations. Therefore, we have systematically searched for serotypes in the GBS data identified regarding maternal colonization, maternal GBS infection, and neonatal/infant disease.

Other important data gaps highlighted by WHO are not covered in this supplement, notably cost-effectiveness analyses. In addition, epidemiological outcomes are not translated into Disability Adjusted Life Years (DALYS). These secondary analyses will be part of later work by WHO and partners on the investments required and other benefits from a maternal GBS vaccine, such as the reduction in maternal antibiotic exposure. The effect on the child’s microbiome is increasingly recognized as important. The final article in the series considers what would be required for a comprehensive investment case regarding GBS, and current vaccine candidates.

## CONCLUSIONS

The lack of etiological data for infections occurring in pregnant women, stillbirths, and infants, in the regions where most births occur, makes the worldwide burden of GBS one of the great “black holes” for public health data worldwide. Other pathogens are also important, including the old foes such as syphilis and gaps for newer foes like HIV/AIDS where stillbirth data have also been neglected. However, among perinatal pathogens, GBS presents specific opportunities, with interventions and potentially high-impact innovation, through maternal vaccination. The following 9 papers outline the most comprehensive data yet, including all relevant outcomes, comprehensive data on serotypes, and extensive attempts to highlight gaps and biases to also inform data improvement. If indeed a significant proportion of the burden occurs before birth, in terms of stillbirths, preterm birth, neonatal encephalopathy, and maternal disease, then this evidence should shift the focus from strategies around the time of birth, such as intrapartum antibiotic prophylaxis , to more upstream prevention such as maternal immunization.

## Notes

***Author contributions.*** The concept of the estimates and the technical oversight of the series was led by J. E. L. and A. C. S. The manuscript was written by J. E. L. with A. C. S. and F. B.-J., and the first authors of articles 2–10 (N. R., J. H., K. L. D., L. M., C. T., M. K.-L.). The GBS Estimates Expert Advisory Group (C. J. B., L. B., C. C., M. G. G., P. T. H., M. I., S. A. M., C. E. R., S. K. S., S. S., A. S.-t. M., J. V.) provided input to the conceptual process throughout, notably on the disease schema and data inputs. All the authors reviewed and provided input to the manuscript.

***Acknowledgments.*** The authors thank Claudia da Silva for administrative assistance and Alegria Perez for coordinating author signatures.

***Financial support.*** This supplement was supported by a grant to the London School of Hygiene & Tropical Medicine from the Bill & Melinda Gates Foundation (Grant ID: OPP1131158).

***Supplement sponsorship.*** This article appears as part of the supplement “The Burden of Group B *Streptococcus* Worldwide for Pregnant Women, Stillbirths, and Children,” sponsored by the Bill & Melinda Gates Foundation and coordinated by the London School of Hygiene & Tropical Medicine.

***Potential conflicts of interest.*** Many contributors to this series have received funding for their research from foundations, especially the Bill & Melinda Gates Foundation, and several from Wellcome Trust, Medical Research Council UK, the Thrasher Foundation, the Meningitis Research Foundation, and one individual from the US National Institutes of Health. Members of the Expert Advisory Group received reimbursement for travel expenses to attend working meetings related to this series. A. S.-t. M. works for the Bill & Melinda Gates Foundation. C. J. B. has served as a member of the Presidential Advisory Committee for Seqirus Inc and of the CureVac Inc Scientific Advisory Committee, as well as undertaken consultancy work for Pfizer Inc. C. C. has received institutional compensation from Novartis for conducting GBS studies. P. T. H. has been a consultant to Novartis and Pfizer on GBS vaccines but received no funding for these activities. M. I. has undertaken sponsored research from Pfizer on pneumococcal disease in adults and from Belpharma Eumedica (Belgium) on Temocilin antimicrobial susceptibility in Enterobacteriaceae. K. L. D. has received funding by the Bill & Melinda Gates Foundation to work on research on GBS serocorrelates of protection to inform vaccine trials, and travel expenses from Pfizer to attend a meeting on an investigator led project on GBS. S. A. M. has collaborated on GBS grants funded by Glaxo Smith Kline and by Pfizer and received personal fees for being member of its advisory committee; he has also collaborated on a GBS grant funded by Minervax. All other authors report no potential conflicts of interest. All authors have submitted the ICMJE Form for Disclosure of Potential Conflicts of Interest. Conflicts that the editors consider relevant to the content of the manuscript have been disclosed.
